# Pilot-Scale Assessment of a Novel Farrowing Creep Area Supplementary Heat Source

**DOI:** 10.3390/ani9110996

**Published:** 2019-11-19

**Authors:** Benjamin C. Smith, Brett C. Ramirez, Steven J. Hoff, Laura L. Greiner

**Affiliations:** 1 Department of Agricultural and Biosystems Engineering, Iowa State University, Ames, IA 50011, USA; bcsmith1@iastate.edu (B.C.S.); hoffer@iastate.edu (S.J.H.); 2 Department of Animal Science, Iowa State University, Ames, IA 50011, USA; greinerl@iastate.edu

**Keywords:** pre-weaning mortality, piglet, microclimate, thermal environment, heat lamp

## Abstract

**Simple Summary:**

Pre-weaning mortality (PWM) causes major economic and productivity losses for the US swine industry. This pilot-scale study evaluated a novel semi-enclosed heated microclimate (SEHM) as a supplementary heat source for farrowing creep areas. Six farrowing cycles (from January to July 2019) were studied in two rooms with 24 farrowing stalls per room. Six SEHMs (each SEHM covers two stalls) were randomly distributed in each room and compared to heat lamps (HLs) for productivity and electricity usage. Data were collected on 113 (SEHM) and 101 litters (HL), and there was no statistically significant difference for average daily gain and weaning weight. There was a tendency for significance of PWM (*p* = 0.08). A significant difference (*p* = 0.02) was noted in the PWM attributed to over-lay mortalities, SEHM = 4.05% (± 0.76%) compared to HL = 6.04% (± 0.78%). The SEHM averaged 3.25 kWh d^−1^ (2.91, 3.59 kWh d^−1^; 95% CI), which was significantly different (*p* < 0.01) from the HL equivalent with 125 W bulbs (6 kWh d^−1^). Based on only electrical savings, payback was estimated at 74 farrowing cycles, or at 12 cycles y^−1^, 6.1 years. The SEHM demonstrated promising pilot-scale results for increasing productivity and decreasing electricity usage compared to conventional HLs.

**Abstract:**

Pre-weaning morality (PWM) is attributed to a poor creep area microclimate and causes major economic and productivity losses for the US swine industry. Piglets need supplementary heat to overcome a high surface area to body weight ratio and minimal thermoregulation. A pilot-scale study was conducted to evaluate a semi-enclosed heated microclimate (SEHM) as a supplementary heat source for farrowing creep areas over six farrowing cycles (from January to July 2019) in two rooms with 24 farrowing stalls in each room. Six SEHMs (each SEHM covers two stalls) were randomly distributed to each room and compared to heat lamps (HLs) for productivity and electricity usage. Data from 113 (SEHM) and 101 litters (HL) showed no significant difference between treatments in average daily gain (*p* = 0.26), 252.4 ± 8.0 g hd^−1^ d^−1^ (SEHM) and 260.3 ± 8.1 g hd^−1^ d^−1^ (HL) and PWM (*p* = 0.08), 9.67% ± 0.82% (SEHM) and 12.04% ± 0.87% (HL). However, a significant difference (*p* = 0.02) was noted in the PWM attributed to over-lay mortalities, 4.05% ± 0.76% (SEHM) compared to 6.04% ± 0.78% (HL). The SEHM electricity averaged 3.25 kWh d^−1^ (2.91, 3.59 kWh d^−1^; 95% CI), which was significantly different (*p* < 0.01) from the HL equivalent (125 W bulb; 6 kWh d^−1^).

## 1. Introduction

Pre-weaning mortality (PWM) is a major economic and productivity challenge for the pig industry. Recent trends for piglets born alive per litter are increasing in the US, while piglets weaned per litter have stayed stable over the past five years [1]; hence, indicating an increasing PWM. PWM is typically greatest during the neonatal phase, or within the first few days after birth in a healthy herd. During this perilous time frame, mortalities may be attributed to multiple causes, with the greatest terminal cause being over-lays (or crushing). There are many secondary causes of over-laying, including infection, starvations, scours, and a poor thermal environment. The thermal environment in which the piglet is born into and lives in during the neonatal phase must meet the specific thermal demands of the piglet to maintain its homeostasis [2]. Thermal needs of piglets are extremely high during the neonatal phase as piglets have a high surface area to body weight ratio and are initially developing their own thermoregulation abilities; thus, they must rely on a microclimate to provide the necessary heat gains to prevent cold stress and maintain their core body temperature. In comparison, microclimate recommendations for piglets range from 32–35 °C dry-bulb temperature (DBT), while for the sows, 19 °C is preferable [3]. Characterizing the thermal environment of piglets is challenging as the thermal environment in the creep area is a combination of multiple factors.

Several parameters can be used to characterize the thermal environment for piglets, namely, DBT, which is the common metric used to quantify the adequacy of piglet microclimate [4,5]. In commercial production, DBT can be incorrectly interpreted as the necessary surface temperatures under a heat lamp (HL) or of a heat mat [3]. This misinterpretation can lead to inadequate thermal environments as it neglects other factors that also impact the effective thermal environment. The parameters that influence sensible heat loss for piglets are DBT, mean radiant temperature, and air velocity, and are considered the most crucial for monitoring [5]. Of these parameters, it has been suggested that the mean radiant temperature could be the most important parameter to monitor in terms of piglet thermal environment [4] as it combines convective and radiative heat losses. The ideal microclimate for piglets is warm, dry, and draft-free, and the specific goal of the thermal environment is providing sufficient heat gains for piglets to avoid dangerous behaviors of seeking the sow’s warmth to gain heat.

To meet the thermal needs of piglets, producers implement a supplementary heat source to create a microclimate within the creep area. The macroclimate of the farrowing room is operated near the upper limit of the sow’s thermoneutral zone due to the risk of cold stressing the piglets. Two of the most common types of supplementary heat sources are HLs and heat mats. Numerous studies have been conducted to evaluate the productivity and electrical energy usage of both heat sources. In general, both perform similarly for average daily gain (ADG) and PWM. However, heat mats have been shown to use less energy compared to HLs [6,7]. The reduction in energy usage of heat mats is attributed to modulated control of all the heat mats in the room based on surface temperature feedback from one DBT or infrared temperature sensor. Recent studies have also suggested that HLs with variable outputs could optimize piglet rectal temperature [8]. The challenge of HLs and heat mat microclimates is that piglets are subjected to air drafts within the creep area. An enclosed microclimate can better protect piglets from cold air drafts, but the lack of supplementary heat control with HLs is still present. Heat lamps are a logical choice for comparing with new technologies in a commercial setting for economic reasons. An added benefit is that HLs are a widely used supplementary heat source featured in other studies related to understanding and improving PWM.

Precision design and control of creep area microclimate have the potential to reduce electrical energy usage and improve piglet productivity. It is, therefore, warranted to examine technologies providing piglets with a draft-free, heated microclimate and that are capable of precision control of the supplementary heat output. The objectives of this pilot study are as follows:

1. Compare thermal environments of a semi-enclosed heated microclimate (SEHM) and heat lamps (HL);
2. Evaluate the production (ADG and PWM) impact of a SEHM;
3. Evaluate the electrical energy usage of a SEHM; and
4. Evaluate the economics of the SEHM based on the pilot study results.

## 2. Materials and Methods

All experimental procedures adhered to guidelines for the ethical and humane use of animals for research and were approved by the Iowa State University Institutional Animal Care and Use Committee (18-216). Mention of trademark, proprietary product, or vendor is for information purposes only. No endorsement implied.

### 2.1. Facility Description

A 1000 head commercial breeding-gestation-farrowing site located in central Iowa (near Ogden, IA) featured nine farrowing rooms, with each room containing twenty-four identical 2.44 (L) × 1.83 m (W) farrowing stalls. Each farrowing stall consisted of a 2.44 (L) × 0.61 m (W) woven wire floor creep area on each side of the sow and a 2.44 (L) × 0.61 m (W) sow area that featured a feeder, cup water, and cast-iron flooring. Each farrowing stall utilized one 125 W incandescent heat lamp (HL) located in the middle of the stall over the creep area as the primary piglet heat source, with a 0.30 × 1.20 m black rubber farrowing mat beneath. An additional 125 W HL was located at the back of the stall in the creep area as a supplement heat source during farrowing and was turned off once the piglets were four days old.

The ventilation system style was negative pressure filtered with an evaporative cooling pad to condition fresh air. Each room featured two variable speed fans (0.46 m; 18 in. diameter) and three single speed fans (0.61 m; 24 in. diameter). One row of 0.71 m (L) bi-directional actuated ceiling inlets distributed fresh air and one liquid propane forced air heater (17.6 kW; 60,000 BTU, Classic 60, LB White, Onalaska, WI, USA) provided supplementary heat. Room air temperature was automatically controlled following standard commercial operation guidelines (Expert V18, Automated Production Systems, Assumption, IL, USA).

### 2.2. Semi-Enclosed Heated Microclimate

**Figure 1:**
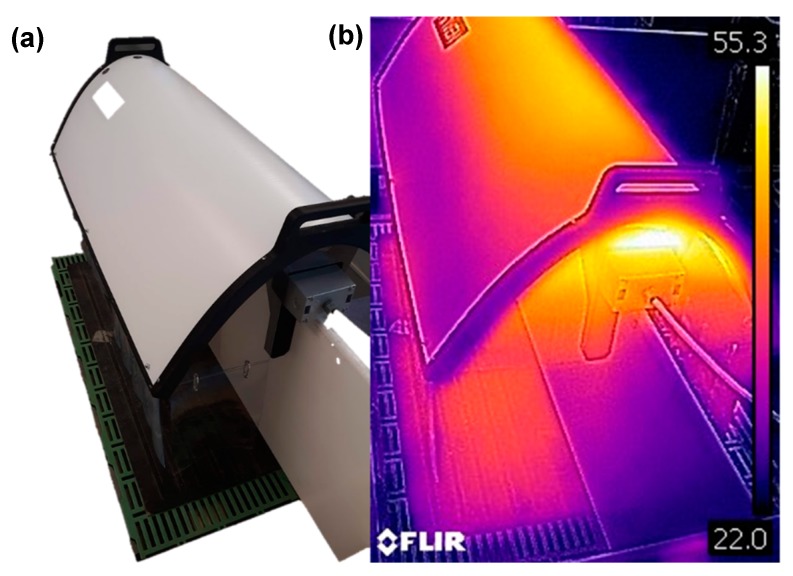
Picture of semi-enclosed heated microclimate (SEHM) for creep area supplementary heat with heating element and enclosure that rest on the creep area divider between two farrowing stalls (**a**). Thermographic image (scale units = °C) of a SEHM at operational conditions (**b**).

The semi-enclosed heated microclimate (SEHM; Haven, FarrPro Inc., Iowa City, IA, USA) tested consisted of a parabolic shaped shield (6.35 mm thick plastic with a 1 mm thick aluminum undercoating) that reflects infrared heat from a horizontal heat element enclosed in a thick-walled glass cylinder. The output of the 600 W ceramic heat element could be varied to provide a custom microclimate. One SEHM covers two farrowing stalls by resting on the creep area divider between the two stalls (Figure 1). The glass cylinder was secured at each end by mounting brackets, with the shield attached to one bracket with a hinge to allow the shield to open and close. Each end of the shield had a clear, rigid plastic end cap, and around the three sides, thick slotted vinyl curtains allow piglets to pass through to access the heated microclimate. Curtains were hung such that the bottom of the curtains were 7.6 cm above the creep area flooring. Each SEHM featured an LED light to provide visible light without shadows underneath the shield. In between each farrowing cycle, the SEHM was power washed using a low-pressure tip and the curtains with a standard high pressure tip. The low-pressure tip was necessary as these beta units were not capable of withstanding a high pressure water jet at close distances.

### 2.3. Instrumentation

A commercially available data acquisition and control (DAQC) system (Fusion, ControlTech, Bondurant, IA, USA) was utilized to condition sensor signals, record measured values, and control the heat output of the SEHMs.

#### 2.3.1. Microenvironment

**Figure 2:**
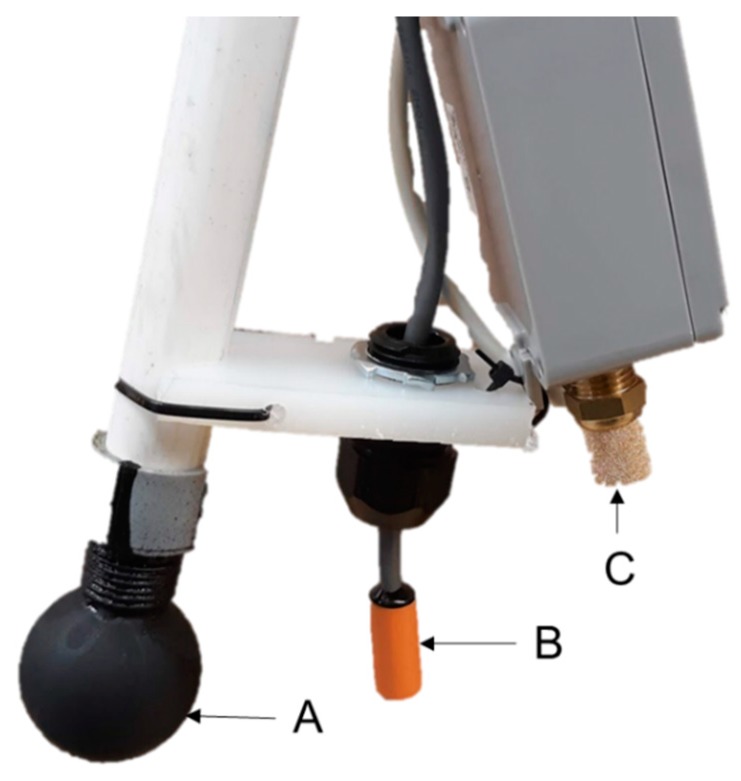
Thermal Environment Sensor Array (TESA) for microclimate instrumentation. Sensors are labeled: (**A**) black globe temperature (BGT); (**B**) dry-bulb temperature (DBT); (**C**) relative humidity (RH).

**Figure 3:**
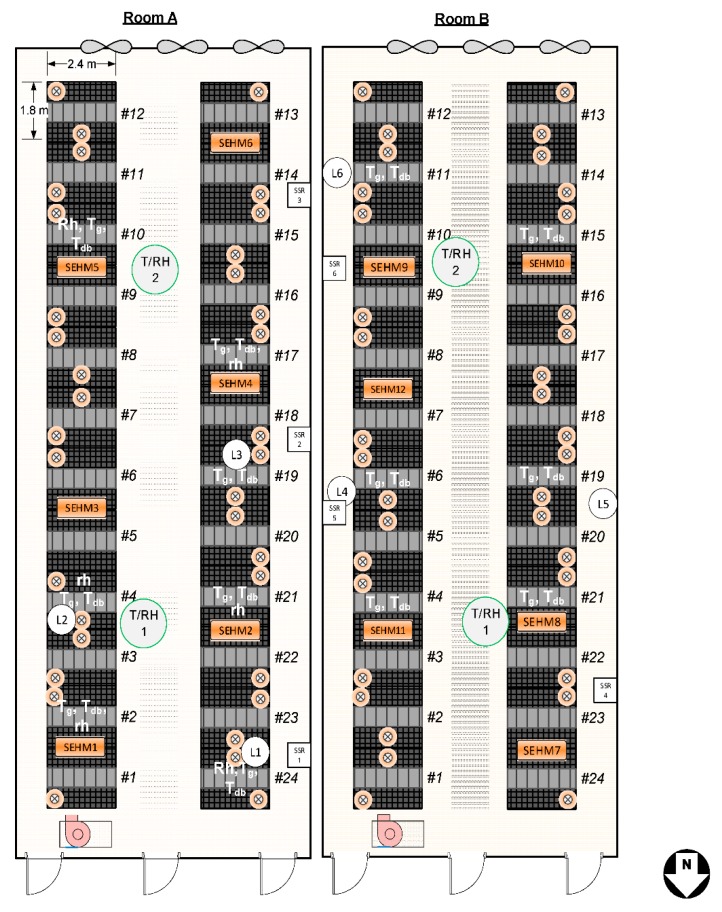
Layout of the two adjacent farrowing rooms (A and B) where the SEHMs were installed. Locations of TESAs for creep area instrumentation and room DBT/RH sensors are identified.

A thermal environment sensor array (TESA; modified from Ramirez et al., 2018 [9]) was constructed for the specific application in this study (Figure 2). Dry-bulb temperature (DBT; F Temp, ControlTech, Bondurant, IA, USA) and relative humidity (RH) sensors (WHT-310, Dwyer, Michigan City, IN, USA) were deployed in key locations, discussed further in this section (Figure 3). A black globe temperature (BGT) sensor was constructed with a DBT sensor (F Temp, ControlTech, Bondurant, IA, USA) placed inside the center of a 5.1 cm diameter copper sphere or a 3.8 cm diameter table tennis ball; both painted flat black. Both types were used due to corrosion of the copper spheres in the high moisture environment, which led to spheres breaking in half. For each type, globes were attached to a 1.27 cm long threaded pipe coupled to a 1.52 cm long PVC pipe with a cord grip on the opposite end to seal the globe enclosure.

Three farrowing stalls in each room assigned to the HL treatment were selected at random to be instrumented with a DBT and BGT sensor. Both sensors were spaced 30.5 cm from the center of the HL and 5.1 cm below the bottom of the HL shield. Also, each TESA was positioned such that the black globes did not contact the divider walls. In room A, two of the three stalls also had an RH sensor installed.

Each SEHM had one DBT sensor installed on one end of the SEHM to serve as feedback for individual control of the heat output. In room A, four SEHMs were instrumented with BGT and RH sensors, and in room B, three SEHMs were instrumented with a BGT sensor as well. All SEHMs with additional sensors (i.e., not for feedback control) were assigned at random. The BGT sensors were installed on one end of the SEHMs such that the black globe was 127 mm into the SEHM and was not touching the divider wall or the end curtain.

#### 2.3.2. Macroenvironment

Two pairs of DBT and RH sensors were placed 1.5 m above the farrowing room floor in the center of the room and 5.4 m from each end wall to monitor room environmental conditions.

#### 2.3.3. Heat Output

**Figure 4:**
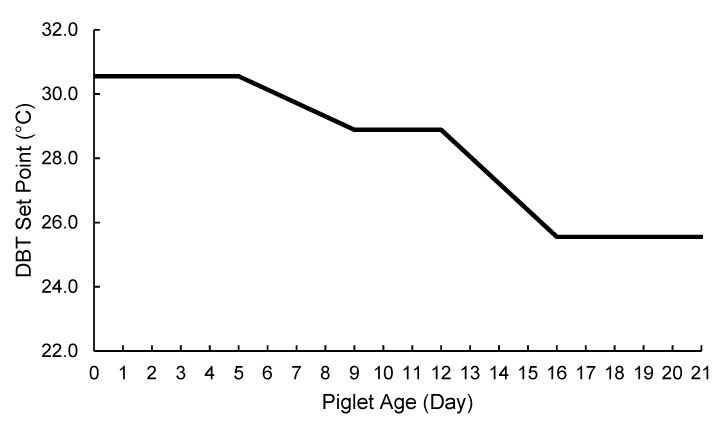
Dry-bulb temperature (DBT) set point curve by piglet age that was used to control the proportional output of the SEHMs.

Heat output for each SEHM was individually controlled using a proportional solid-state relay (RM1E23V25, Carlo Gavazzi Inc., Buffalo Grove, IL, USA) and a solid-state true RMS current clamp was used to monitor electrical current (Model CCT70-100, Dwyer Michigan City, IN, USA). The control logic applied a sliding proportional output based on a DBT set-point curve that decreased with increasing piglet age. Essentially, when DBT was below the DBT set-point, output was 100%, and as DBT approached and exceeded the set-point, output decreased, with output at 0% when DBT was 2.78 °C above the set-point. This control logic began once the youngest litter in the two stalls farrowed (Figure 4). Maximum heat output was limited to 425 W by capping the proportional percentage due to amperage constraints associated with farrowing room electrical utilities. Heat lamps were always operational (constant energy usage), and the height of HL above the creep area was adjusted by the farm staff as needed to control the farrowing mat temperature.

#### 2.3.4. Sensor Verification

Once all sensors were installed in the farrowing rooms, DBT and RH sensor performance were verified using a portable hygrometer (Model HMI41, Vailsala, Vantaa, Finland), the RH sensor was calibrated with a salt chamber system (Model HMK15, Vaisala Vantaa, Finland). A two-point calibration was created by collocating sensors in a cardboard box and allowing the portable hygrometer to achieve steady-state at two state points: (1) room air temperature and (2) at the incoming ventilation air temperature, which was lower than room temperature. The incoming air state was achieved by placing the box of collocated sensors in the ceiling inlet air jet. Any DBT sensors exceeding ±1.1 °C and any RH sensors exceeding ±5% RH of the portable hygrometer were replaced with new sensors.

Each current clamp was calibrated using a portable power meter (Model 1735, Fluke, Everett, WA, USA) at the start of the first and second farrowing cycle. No significant differences were noted in the current clamp calibrations warranting future calibrations were not needed with each subsequent farrowing cycle.

### 2.4. Data Recording and Processing

All data were recorded by the DAQC using a sparse sampling method of a minimum change threshold of 0.23 °C, 0.5% RH, or 0.5 A (for each respective sensor) or at a 5 min interval if the minimum change threshold had not occurred.

Data were downloaded at the conclusion of each farrowing cycle for all micro-/macro-environment sensors and the SEHM control data. Custom software (Python 3.7, Python Software Foundation, Wilmington, DE, USA) developed in an integrated development environment Project Jupyter (2015), processed and organized the data [10]. The date range for each creep area TESA was the date of farrowing at midnight to 08:00 on the day of weaning. Mean radiant temperature was calculated using BGT and DBT assuming only natural convection [11]. Data were averaged across all SEHM, HL, and room locations to determine average DBTs and RHs for the SEHM, HL, and room for each room for all six farrowing cycles.

### 2.5. Piglet Productivity

Two rooms were selected to perform the pilot-scale study. Each room was randomly allocated 12 farrowing stalls with 125 W heat lamps (HL treatment) and 12 farrowing stalls with SEHM (Figure 3; SEHM treatment). Commercial sows (Landrace × Yorkshire) and piglets ((Landrace × Yorkshire) × Duroc; PIC genetics) were utilized in the study. The minimum number of litters needed in the study was determined using a power analysis for PWM as the smallest anticipated response. The standard deviation was estimated from similar studies [7], nominal power was set to 85%, and the detection level was set to 1.25%. The resulting minimum was determined to be 94 litters per treatment.

The sows were limit fed 1.8 kg twice daily from day 112 of gestation until day four of lactation. The lactation diet was corn and soy bean meal-based diet that met or exceeded the National Research Council (NRC, 2012) requirements [12]. At day 4 of lactation, the sows were transitioned to an ad libitum feeding program that was accomplished using an automatic feed delivery system that delivered feed twice daily. The feeder for each sow had a holding capacity of 8.2 kg. Sow feed disappearance was considered as a response variable, but due to the limitations of the commercial site for accurate, repeatable measurements of feed delivered to the sows, it was not measured.

Litter weight and mortality data were collected for six farrowing cycles (January 2019 to July 2019). Litters were weighed in the morning between days 1 and 3 (after litters were cross-fostered to standardize litter size, i.e., number of piglets) and between day 17 and 19 (prior to weaning). Cross-fostering occurred between 24 to 48 h after farrowing (farm’s target was 13 piglets per sow), and if piglets were cross-fostered across treatments, the litter was removed from the trial. Litter weights were collected by the research team using a portable litter scale (WayPig^®^ Portable Litter Scale, Raytec, Ephrata, PA, USA). Mortality weights were collected using a bucket scale (Agri-Pro, Iowa Falls, IA, USA) by both farm staff and the research team throughout the study. Prior to weighing, the litter scale was verified against a standard weight (22.7 kg; NIST traceable), and the bucket scale was verified once a week with the same standard weight. Mortality data were gathered from the farm’s production record system (Porcitec 2017, Agritec Wheatland, IA, USA), including mortality date and cause of death. To ensure data quality and consistency, data pertinent to the litter, including date of birth (DOB), mortalities, cross-fosters, total born live (TBL), and parity, were manually transcribed from the sow farrowing records by the research team immediately prior to weaning. The terminal cause of death was recorded for the mortalities to remove bias in diagnosing potential underlying causes that led to death, such as infections.

#### Data and Statistical Analysis

Pre-weaning mortality was calculated using Equation (1). The number of mortalities and the dates were verified using the sow farrowing records and the mortality weight records before data analysis. Consensus across all three data sources for the number of mortalities, number of pigs in the litter, and date of the mortalities was needed to keep a litter enrolled in the study. The over-lay percentage was calculated using a similar equation to that in Equation (1), except the numerator was the number of over-lay mortalities.

(1) $$PWM\prime =\frac{Number\;of\;mortalities}{TBL+FST}$$

where, *PWMʹ* = pre-weaning mortality with fosters; *TBL* = total born live; *FST* = number of piglets added or removed per litter (at cross-fostering)

The statistical procedures of JMP 14 (SAS Inc., Cary, NC, USA) were utilized to analyze the data using Mixed Models. A backward model selection approach was used with a cutoff significance of *p* = 0.80 and an *α* = 0.05. Data were checked for normality between treatments and outliers. Fixed effects for the models included heat source, health status at first weight, sow parity group, TBL, FST, number of pigs in the litter when weighed, average piglet weight at first weigh, full factorial of interactions of all fixed effects, and the farrowing cycle was considered as a random effect. Sow parity groups were classified as young (P1 and P2), prime (P3 to P5), and geriatric (P6 and above).

### 2.6. Electrical Energy Usage

Electrical energy usage of the SEHMs was calculated from the measured current and control percentage. Electrical current was measured continuously using the aforementioned current clamps and DAQ system using a sparse logging approach. Similar studies have shown this method of continuous current monitoring to be accurate for calculating electrical energy usage [13]. A voltage (mean R^2^ = 0.99; RMSE = 2.40 V_AC_) and power factor (mean R^2^ = 0.95; RMSE = 0.04) relationship was generated to correspond with the control percentage range (0% to 100%) using the portable power meter, when the current clamps were verified described in the sensor verification section. These relationships were necessary as the proportional solid-state relays altered the power factor of the circuit and the voltage the heat element received as a function of the control percentage. The voltage and power factor were calculated for each timestamp when a current was recorded by the DAQ, using the described relationship and assumed constant to the next timestamp. The power was then calculated utilizing Ohm’s Law multiplied by the power factor, single phase 120 V circuit. The duration between timestamps was used to calculate kWh. Heat lamp electricity usage was verified using the power meter, and the equivalent electricity usage for the HLs was calculated as the verified kWh usage multiplied by the duration that the SEHM had power supplied to it.

### 2.7. Pilot-Scale Economics

The payback period for an SEHM was estimated to assess the economic feasibility and provide insight to producers on the potential value of this device. Electrical energy usage for HLs and SEHMs was used as the input as it is repeatable across production systems with a similar control setup. Piglet productivity varies considerably and is challenging to define costs associated with differences in average daily gain and piglets weaned per litter. Electricity cost for Iowa was assumed to be 0.10 USD kWh^−1^ [14]. The capital cost of the SEHM was assumed at $400 per unit; this includes the heating element and control system. The capital cost of two HLs was assumed to be $14.50. For utilities and other installation infrastructure, it was assumed to be the same for both SEHM and HL.

The net income for the SEHMs within a commercial production system was calculated based on supply costs and replacement assumptions. For the net profit analysis, the following was assumed, curtains would have to be replaced semi-annually ($25 for a set of curtains), and for HLs, bulbs would be replaced three times per year, for a total of six bulbs per year (two HLs; $1.99 bulb^−1^). Wean pig market price ($46.34 [15]) was collected from the USDA as the average of the first and second quarter national prices.

## 3. Results and Discussion

For six farrowing cycles, 113 litters (SEHM treatment) and 101 litters (HL treatment) were farrowed and weaned for this analysis. There were 25 SEHM litters and 37 HL litters excluded from this study due to sow mortality, sow health, and piglet mortality data quality.

### 3.1. Overview

**Figure 5:**
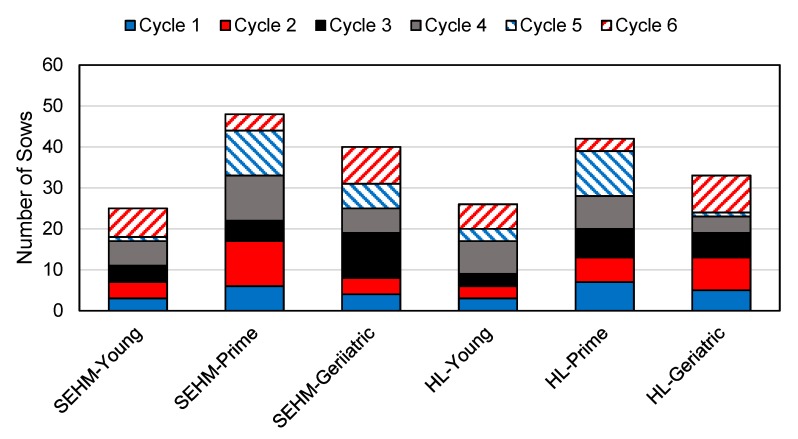
Number of sows by sow parity group (young, prime, and geriatric) and heat source treatment (SEHM and HL) enrolled in this study. Sow parity groups are further distinguished by farrowing cycle, depicted by fill within bars.

Parity distribution by treatment is shown in Figure 5 and demonstrates a balanced distribution of sow parity groups (SPGs) across treatment groups. During this study, the two rooms averaged a TBL of 13.6 piglets and the farm was negative for Porcine Reproductive and Respiratory Syndrome Virus (PRRSv). The following sections address each objective of this study.

### 3.2. Temperature Profiles

**Figure 6:**
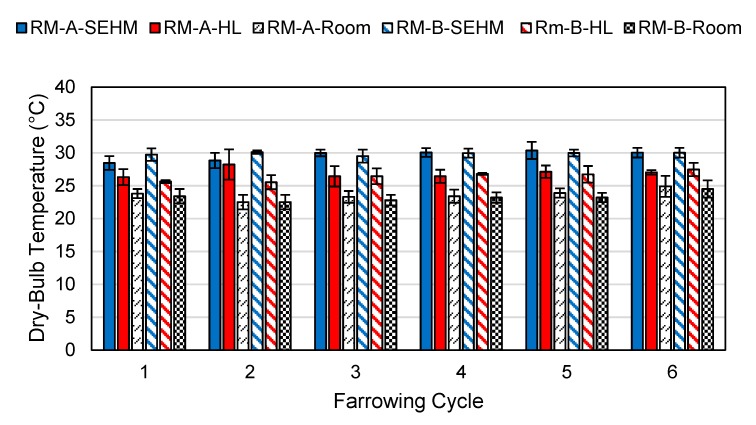
Average dry-bulb temperature of the creep area microclimate by heat source and room macroclimate (A and B) by farrowing cycle. Error bars represent one standard deviation of the mean.

**Figure 7:**
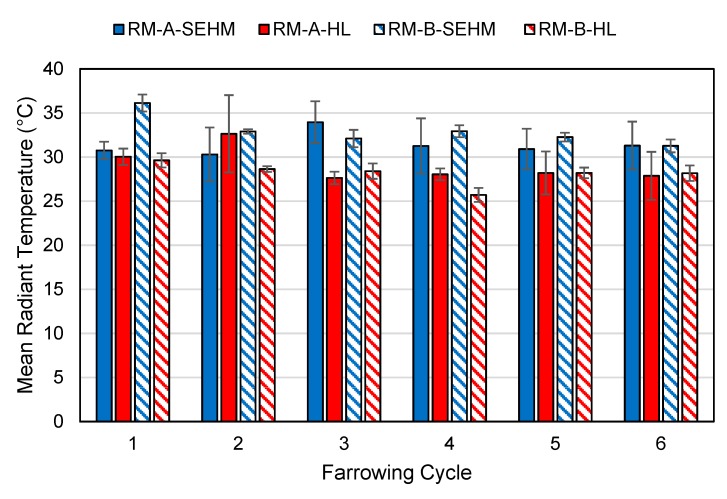
Mean radiant temperature of the creep area microclimate by heat source and farrowing cycle. Error bars represent one standard deviation of the mean.

**Figure 8:**
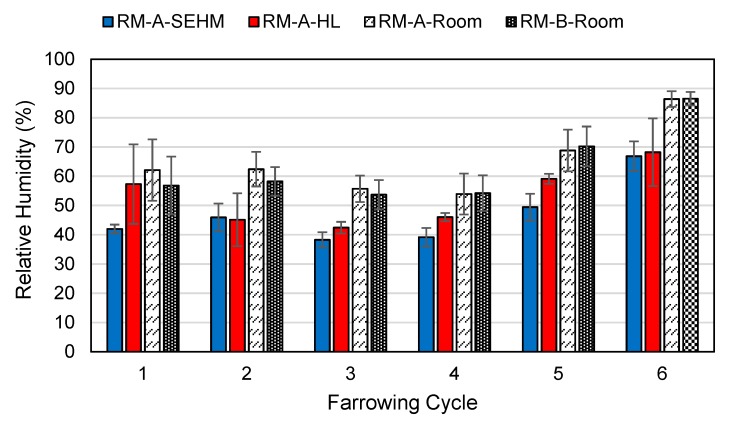
Relative humidity of the creep area microclimate in room A and the average relative humidity of room A and B macroclimate.

One RH sensor failed the verification tolerance, and no DBT sensors failed verification during the study. Average mean radiant temperature (MRT) under the SEHM was 32.3 °C compared to the HL average MRT of 28.6 °C. Average DBT under the SEHM was 29.8 °C compared to the HL average of 26.7 °C. There was a trend observed for the SEHM’s creep area having a warmer DBT and MRT for both rooms across all farrowing cycles in comparison to the HL treatment, as shown in Figure 6 and Figure 7. As expected, RH was lower under SEHM compared to HL, since DBT was greater, as shown in Figure 8. The perceived air quality within the SEHM was comparable with the room conditions.

### 3.3. Pre-Weaning Mortality

**Figure 9:**
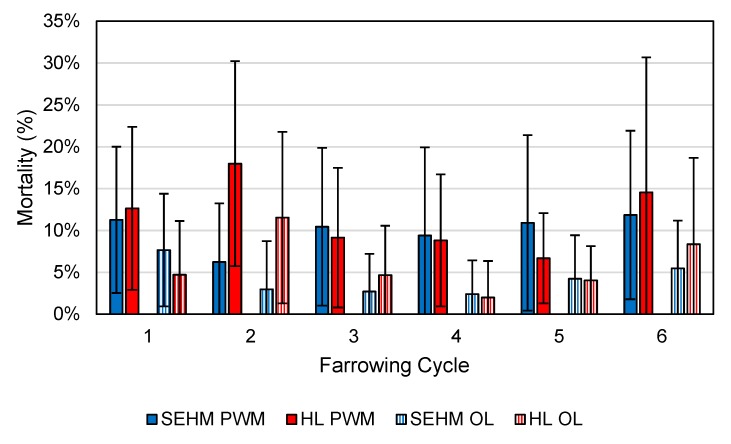
Pre-weaning mortality (PWM) and over-lay mortality (OL) percentages over six farrowing cycles for the two heat source treatments, SEHM and heat lamp (HL). Error bars represent one standard deviation of the farrowing cycle mean. This data represents the raw results, and thus, no statistical analysis between cycles was performed.

For reference, all nine farrowing rooms had an average PWM of 10.76% (SE = 0.70%) with an over-lay (OL) percentage of 4.87% (SE = 0.46%). The PWM and OL percentages of the two rooms in this study are shown in Figure 9. Following the completion of the second cycle the maximum heat output of the SEHM was lowered by 75 W due to mortality timing concerns to a new maximum output of 350 W. Numerous observations were made by both the researchers and the farm staff noting piglets lying outside the SEHM units throughout lactation suggesting the heat output was too high.

The final statistical model used to analyze overall PWM comprised of the farrowing cycle as a random effect as well as the following fixed effects and interactions: treatment, sow parity group (SPG), TBL, FST, and treatment * SPG, SPG * FST. The statistical model for OL had the same fixed effects as the PWM model and an additional interaction term: treatment * FST. The comparison of treatments is shown in Table 1. The significant effects on PWM were SPG, TBL, FST, and treatment * SPG. Additionally, significant effects on OL mortality were treatment, SPG, TBL, FST, and treatment * SPG. The results of a Tukey HSD comparing the interactions of treatment and SPG on PWM and OL mortality are shown in Table 2.

Greater PWM was noted in SPG-Geriatric for both treatments and was not attributed to outliers in the dataset. The SPG and treatment interaction lacked the necessary sample size to detect differences between the interaction groups. Albeit, the magnitude of the difference between SPG-Geriatric and SPG-Young plus SPG-Prime together, was large, SPG-Geriatric was the only SPG significantly greater than all others, showing there was an increased benefit of the SEHM treatment with older sows (Parity 6 and greater). Similar studies have presented solely PWM difference by treatment and did not explore the interactions of sow parity and piglet heat sources when comparing HLs and heat mats [6,7]. The inclusion of sow parity in this study offers an insight into the overall impact of the SEHM in relation to sow parity as parity is a well-known factor of PWM in a commercial setting.

### 3.4. Average Daily Gain

**Figure 10:**
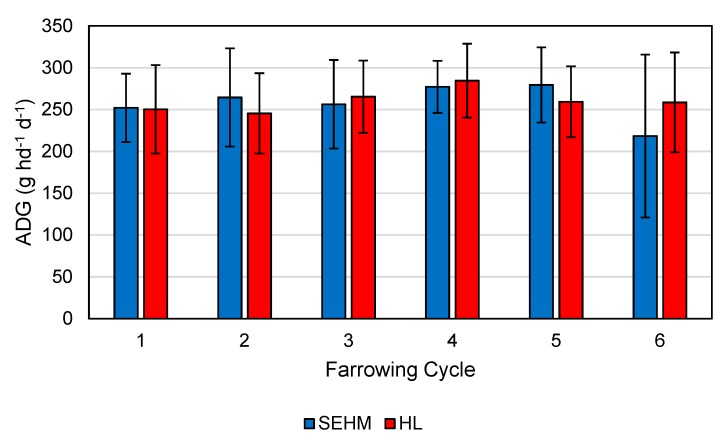
Average daily gain (ADG) across SEHM and HL treatments by farrowing cycle. Error bars represent one standard deviation of the farrowing cycle mean. This data represents the raw results, and thus, no statistical analysis between cycles was performed.

Mean ADG for all litters included in the study was 260 g hd^−1^ d^−1^ (SE = 3.82), with the cycle average by treatment shown in Figure 10. No significant differences were noted in average piglet start weight (*p* = 0.40) 1.59 ± 0.02 kg (3.54 ± 0.05 lb) and average piglet end weight (*p* = 0.46) 5.82 ± 0.06 kg (12.84 ± 0.13 lb).

The final statistical model for ADG included treatment, SPG, average starting weight, number of pigs at the start, and treatment*SPG as fixed effects and farrowing cycle as a random effect. The ADG for SEHM was 252 g hd^−1^ d^−1^ and HL was 260 g hd^−1^ d^−1^, and the difference was not significant (*p* = 0.26). Similar studies have reported ADG from 230 to 246 g hd^−1^ d^−1^ [6] and from 220 to 224 g hd^−1^ d^−1^ [7]. These studies recorded weight gain with different methods compared to this study, which could be a cause for the difference, as well as differences in age at weaning and the genetic lines utilized in each study. The slightly lower ADG for the SEHM was expected as the average number of piglets per litter after cross fostering was higher 13.8 piglets for SEHM and 13.4 piglets for HL though not significant (*p* = 0.12) still would have created higher competition during feeding.

### 3.5. Electrical Energy Usage

**Figure 11:**
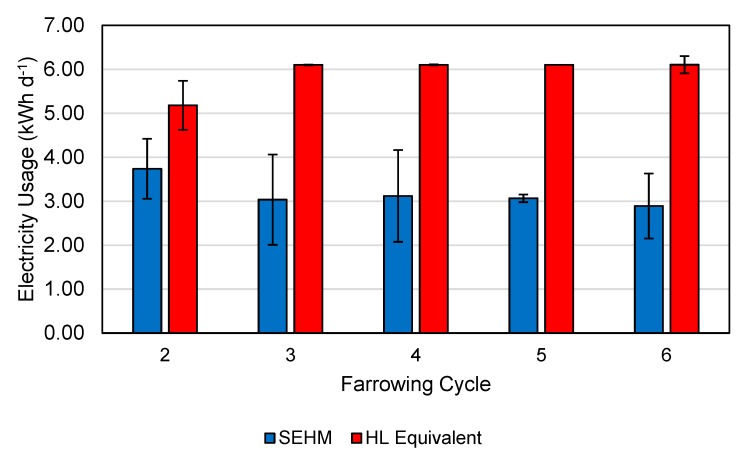
Electricity usage per day for SEHMs over five farrowing cycles utilized for the energy usage analysis. Error bars represent one standard deviation of the mean. Only the units with both litters farrowing on the same day were utilized for comparison. Note, farrowing cycle 2 had a higher maximum output setting than the later cycles, and the lower heat lamp equivalent is due to power outage issues on the farm late in the cycle.

Only the last five farrowing cycles were considered for calculating electricity usage since the SEHMs in the first cycle featured a different shield design that was replaced at the end of the cycle. For standardization, only electrical data from SEHMs that had both litters farrow on the same date were used. A total of 21 units across the five cycles (February to July) were analyzed for energy usage with the average kWh d^−1^ shown in Figure 11. Average SEHM energy usage was 3.25 kWh d^−1^ (2.91, 3.59 kWh d^−1^; 95% CI), which was significantly different (*p* < 0.01) compared to the HL equivalent with 125 W bulbs (6 kWh d^−1^). This is a 59% reduction in electricity over a 19 d duration. Following the completion of the second farrowing cycle, the maximum heat output was lowered by decreasing the proportional percentage maximum. This was decided upon based upon the researcher’s and farm staff’s observations of piglets not utilizing the SEHM in late lactation and concerns for mortalities during that time. Heat mats have been reported to reduce electricity by 36% compared to HLs with 125 W bulbs [7]. The greater electricity reduction noted in this study was most likely attributed to the control system, with each SEHM being individually controlled. The heat mat system referenced above had half of one room (20 farrowing stalls) controlled from one control unit temperature sensor. The reduction of electricity used in this study demonstrates the potential for precision livestock systems to improve the overall efficiency of the system.

### 3.6. Pilot-Scale Economics

The payback period for a SEHM (only the capital cost of $400) was based on the electricity usage reduction and was estimated to be 6.1 years or 74 farrowing cycles, for a 24 farrowing stall room. This payback is based on an average lactation length of 19 days. A production system operating with 16 farrowing cycles per year, the payback period is 4.6 years. A similar study evaluating HLs and heat mats on a commercial sow farm noted a payback period for heat mats of 57 farrowing cycles [7]. This study evaluated the energy usage at the room level with a single power meter for all the heat elements in half of the farrowing room. A payback based solely on the electrical savings is more reproducible across different production systems as long as the same control system is utilized. Farrowing management style (e.g., length of lactation, synchrony of farrowing) will also impact the economic outcome. If both litters were not the same age, the SEHM’s electrical usage would increase because the curve would be delayed starting until the youngest litter is born.

The reduction in overall PWM by 2.33% resulted in a net income from additional pigs weaned of $29.37 per cycle for the SEHM. Combined with the electricity savings and the operating costs (cost of replacing curtains minus the cost of HL bulbs; −$38.06 per year), the net profit per cycle for a SEHM was $32 or $380 per year. Under the conditions of this pilot-scale study, the cooperating farm in this study achieved a net income from the SEHM over the six cycles (*n* = 71) of $2248. This does not include the capital cost of the SEHMs and the control system. These net profit values for the SEHM are sensitive to the wean pig market price as it comprises the majority of the value. This estimate should be updated as wean market prices change. Further, the PWM difference between SEHM and HL will vary across production systems as management also substantially contributes to this value. Large PWM variability is a known challenge in pig production [16], thereby creating some uncertainty in this estimated value.

## 4. Conclusions

A pilot study was completed to characterize microclimate profile, production impact, energy usage, and economic impact of a novel semi-enclosed heated microclimate (SEMH). Two rooms of 24 farrowing stalls were randomly assigned to either heat lamps (HL) or SEHMs as the primary heat source and tested over six farrowing cycles. The key results are listed below.

- Average mean radiant temperature (MRT) under the SEHM was 32.3 °C compared to the HL average MRT of 28.6 °C.
- Average dry-bulb temperature (DBT) under the SEHM was 29.8 °C compared to the HL average DBT of 25.6 °C.
- There was no statistically significant difference for average daily gain (*p* = 0.26) and weaning weight (*p* = 0.46).
- There was a tendency for significance for PWM (*p* = 0.08).
- A significant difference (*p* = 0.02) was noted in the PWM attributed to over-lay mortalities, SEHM = 4.05% (± averaged 3.25 kWh d^−1^ (2.91, 3.59 kWh d^−1^; 95% CI), which was significantly different (*p* < 0.01) from the HL equivalent with 125 W bulbs (6 kWh d^−1^).
- Based on only electrical savings, payback was estimated at 74 farrowing cycles, or at 12 cycles y^−1^, 6.1 years.

The SEHM provided a warmer microclimate compared to conventional HLs, thereby meeting the thermal needs of neonatal piglets. No difference was noted in the growth rate, suggesting the creep area heat source has a limited impact on the sow and her milk production; however, a covered creep area will enable reduced farrowing room temperatures, which may improve sow body condition. The majority of the overall PWM difference is explained through the difference in over-lay mortalities. Lower electrical energy usage was attributed to the individualized control of each SEHM.

This pilot-scale study showed a positive impact of the SEHMs on piglet productivity. The direct application of these results to commercial production systems may be limited since the heat source treatments were randomized within each room; thus, introducing unique management for each heat source. This study also did not consider the impact the heat source has on the sow, such as feed intake and body weight change. This effect will also have a large impact on the overall productivity of the production system as the sow’s subsequent litter size and lactation performance can be impacted by the current lactation.

## Figures and Tables

**Table 1:** Comparison of average (standard error; SE) pre-weaning mortality (PWM) and over-lay (OL) percentage between semi-enclosed heated microclimate (SEHM; *n* = 113) and heat lamp (HL; *n* = 101) for six farrowing cycles. Levels not connected by the same superscript letter are significantly different (*α* = 0.05).

Treatment	PWM (SE)	Over-Lay (SE)
SEHM	9.67% (0.82) ^a^	4.05% (0.76) ^a^
HL	12.04% (0.87) ^a^	6.04% (0.78) ^b^

**Table 2:** Interaction of treatments (SEHM and HL) and sow parity group (SPG) for PWM and over-lay percentage. Levels not connected by the same superscript letter are significantly different (*α* = 0.05).

Treatment	SPG	PWM (SE)	Over-Lay (SE)
SEHM	Young	9.07% (1.86) ^a^	4.10% (1.30) ^a^
	Prime	9.78% (1.33) ^a^	2.97% (1.00) ^a^
	Geriatric	10.16% (1.48) ^a^	5.07% (1.10) ^a^
HL	Young	7.78% (1.86) ^a^	2.87% (1.29) ^a^
	Prime	9.10% (1.43) ^a^	4.10% (1.07) ^a^
	Geriatric	19.23% (1.64) ^b^	11.14% (1.18) ^b^
